# Biological and Pharmacological Potential of Xylitol: A Molecular Insight of Unique Metabolism

**DOI:** 10.3390/foods9111592

**Published:** 2020-11-02

**Authors:** Vishal Ahuja, Markéta Macho, Daniela Ewe, Manoj Singh, Subhasish Saha, Kumar Saurav

**Affiliations:** 1 Department of Biotechnology, Himachal Pradesh University, Shimla 171005, India; vahuja3@gmail.com; 2 Laboratory of Algal Biotechnology—Centre Algatech, Institute of Microbiology of the Czech Academy of Sciences, 37901 Třeboň, Czech Republic; macho@alga.cz (M.M.); ewe@alga.cz (D.E.); saha@alga.cz (S.S.); 3 Faculty of Science, University of South Bohemia, 37005 České Budějovice, Czech Republic; 4 Department of Biotechnology, Maharishi Markandeshwar (Deemed to be University), Mullana 133207, India; manoj.singh@mmumullana.org

**Keywords:** xylitol, nutritive sweetener, anti-cancer, anti-inflammatory, respiratory tract infection, cardiovascular diseases

## Abstract

Xylitol is a white crystalline, amorphous sugar alcohol and low-calorie sweetener. Xylitol prevents demineralization of teeth and bones, otitis media infection, respiratory tract infections, inflammation and cancer progression. NADPH generated in xylitol metabolism aid in the treatment of glucose-6-phosphate deficiency-associated hemolytic anemia. Moreover, it has a negligible effect on blood glucose and plasma insulin levels due to its unique metabolism. Its diverse applications in pharmaceuticals, cosmetics, food and polymer industries fueled its market growth and made it one of the top 12 bio-products. Recently, xylitol has also been used as a drug carrier due to its high permeability and non-toxic nature. However, it become a challenge to fulfil the rapidly increasing market demand of xylitol. Xylitol is present in fruit and vegetables, but at very low concentrations, which is not adequate to satisfy the consumer demand. With the passage of time, other methods including chemical catalysis, microbial and enzymatic biotransformation, have also been developed for its large-scale production. Nevertheless, large scale production still suffers from high cost of production. In this review, we summarize some alternative approaches and recent advancements that significantly improve the yield and lower the cost of production.

## 1. Introduction

Xylitol (1,2,3,4,5-pentol), a natural five-carbon sugar alcohol, is among the most valuable microbial products commonly used as a sweetening agent. Xylitol was first isolated from the bark of beech trees in 1890. The application of xylitol varied widely from food, nutraceuticals, beverage to pharma industries, making it one of the top 12 global bio-products [1,2] with a rapidly growing market share, which is expected to cross USD 1.14 billion by 2023. Xylitol is considered as a “Generally Recognized as Safe” (GRAS) additive by the Food and Drug Administration (FDA) and as a low-calorie sweetener by more than 35 countries [1,3]. The chemically less reactive nature of xylitol offers unique pharmacological properties for the treatment of various ailments. The increasing global demand due to greater awareness of the useful properties of xylitol has led to the steep rise in its production. Tate & Lyle PLC (London, UK), Cargill, Incorporated (Wayzata, MN, USA), E. I. du Pont de Nemours and Company (Wilmington, DE, USA), Danisco (DuPont, Copenhagen, Denmark), Novagreen Inc (Edmonton, AB, Canada), Futaste Pharmaceutical (Yucheng, China), Yucheng Lujian (Shandong, China) and Hangzhou Shouxing BioTechnology Co. Ltd. (Yuhang, China) are the dominant leading developers and shareholders for xylitol production.

Almost 70% of the market share globally accounts for the manufacturing of chewing gums and confectionery products (Table 1) [4]. It has also been claimed by the European Food Safety Agency that xylitol chewing gum reduces the risk of caries in children [5]. Several reviews have been published in recent years with xylitol application in pharmaceuticals (especially dental care) and its prospects of production. Herein, we aim to review the development of production methods with time including chemical as well as biological routes together with its application in pharmaceutical industry for the treatment/prevention of various diseases. We also tried to mention other strategies, involving strain improvement in brief with more emphasis on substrates and choice of bioprocess to achieve better productivity.

## 2. Xylitol Production

**Figure 1:**
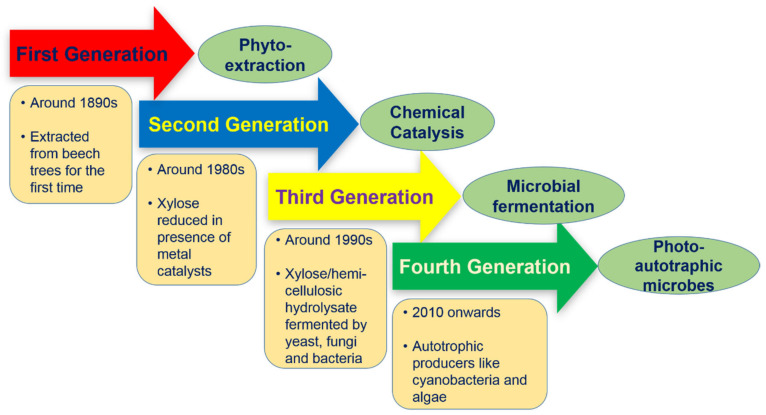
Generations of xylitol production from various substrate and catalytic agents. The journey began with the extraction of xylitol from wood (first generation) followed by catalytic reduction of xylose or xylose rich hydrolysate by metal catalysts (second generation). Bioprocessing, which uses photoautotrophic microorganisms developed/engineered to produce xylitol, predominates from the third generation onwards.

The journey of sustainability begins with the shifting of commercial production processes to become economically and environment friendly. Like biofuels, xylitol production methods can be classified into different generations based on the sources and catalytic agents used for substrate transformation (Figure 1). The first two generations are the conventional as well as primitive methods for xylitol production which include extraction from plant sources (woods, fruits and vegetables) followed by the catalytic reduction of xylose. Both approaches suffer with high cost of production due to the necessity of specialized and expensive equipment, long purification steps, catalyst deactivation, and finally, the huge consumption of energy. Hence, alternative methods have been developed for xylitol production in more economic and ecofriendly manner.

### 2.1. First Generation (Phyto-Extraction)

Plants are one of the most prominent natural producers of xylitol. Until the 1960s, extraction from wood was the primary and probably the sole source for xylitol due to lack of research [3]. Later, it was also extracted from vegetables and fruits by solvent extraction (Table 2). The maximum concentration of xylitol has been reported from yellow plum, and greengages comprise about 1% of dry weight [6,7,8,9].

### 2.2. Second Generation (Catalytic Reduction)

During 1970s, the first industrial production was initiated in Finland by developing mass production of D-xylose via chromatographic separation from various woody hemicelluloses. Production was followed by catalytic reduction of D-xylose to xylitol under high hydrogen pressure and temperature. These methods employed several purification steps due to the requirement of pure d-xylose feed for catalytic reduction [4]. Briefly, chemical catalysis includes the reduction of xylose under high pressure and temperature (80–140 °C and up to 800 psi pressures) in the presence of a metal catalyst, Raney Nickel [10]. Moreover, some other metal catalysts, including ruthenium, titanium or a mixture of multiple metals, have also been used for a catalytic reduction under varying temperatures. Catalytic reduction yielded up to 60% conversion of xylose, depending on the purity of the substrate and reaction conditions [11,12]. Recently, xylose is reduced in the presence of 5 wt.% simple silica-supported monometallic cobalt-Co/SiO_2_-catalyst at 150 °C, under 5 MPa of hydrogen for 30 min. The process yielded up to 77% xylitol, which increased to 98% under optimized operating conditions [13]. However, the metal catalysis has been under scrutiny due to its high operating cost and poor environmental sustainability.

### 2.3. Third Generation (Microbial Fermentation and Enzymatic Transformation)

**Figure 2:**
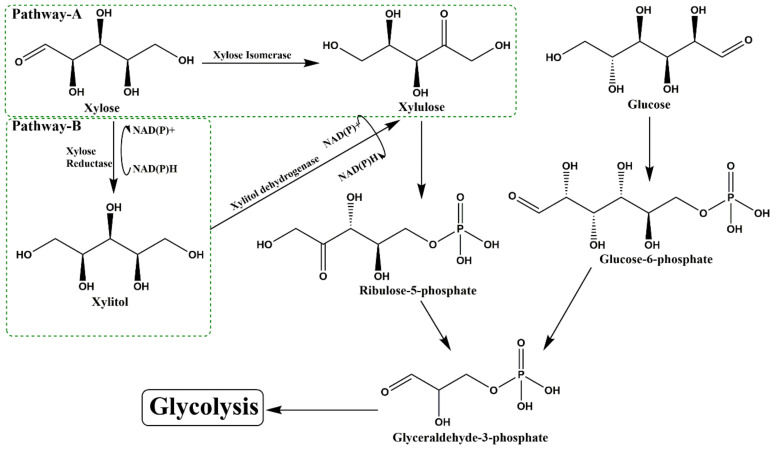
Xylose metabolism in (**A**) Xylitol non-producers and (**B**) Xylitol producer. Prokaryotes are able to oxidize xylose directly to xylulose in the presence of xylose isomerase (Pathway A) while in eukaryotes, this conversion proceeds with xylitol as an intermediate product (Pathway B).

Similar to plants, microbes are natural xylitol producers, which reduce xylose to xylitol by xylose reductase enzyme. Metabolic fate of xylose in microbes operates via two different pathways (Figure 2).

Pathway A: Among prokaryotes, intracellular xylose is directly transformed to xylulose by xylose isomerase (Enzyme commission Number; EC 5.3.1.5).

Pathway B: In eukaryotes, the conversion of xylose to xylulose is a two-step process. NAD(P)H/NADH-dependent xylose reductase (1.1.1.307; XR) reduces intracellular xylose to xylitol, which further oxidizes to xylulose by NAD^+^-dependent xylitol dehydrogenase (EC 1.1.1.9, XDH).

Further metabolism of xylulose is a common phenomenon for both eukaryotes and prokaryotes. Xylulose participates in the pentose phosphate pathway and glycolysis for energy generation [14]. Thus, the microbes with active xylose reductase pathway are possible biocatalysts for xylitol production at a large scale. Although “pathway B” predominates in fungi, e.g., *Aspergillus niger* [15], yeast, e.g., *Candida guilliermondii* [16], *Pichia spitis* [17], *Pichia caribbica* [18] and *Scheffersomyces amazonensis* [19], and dairy yeast, e.g., *Kluyveromyces marxianus* [20], but few bacteria, e.g., *Enterobacter* sp. [21], *Corynebacterium*, *Enterobacter* [22], *Bacillus* and *Pseudomonas* [23], were also reported for xylitol production (Table 3).

Around 960 wild yeast strains were isolated from soil, wood and insect larvae and termites and compared for xylose consumption. Based on the higher xylose consumption, 42 yeasts strains were selected for metabolic profiling and microscale fermentation of sugarcane bagasse hydrolysate. Xylitol yield (Y_P/S_) from defined medium and sugarcane bagasse hydrolysate varied from 0.25 g/g–0.34 g/g (defined medium) to 0.41 g/g–0.60 g/g (sugarcane bagasse hydrolysate). Yeast strain with optimum xylitol production was identified and characterized as *M. guilliermondii* B12. The newly identified strain was compared with earlier known yeasts *M. guilliermondii* A3, *Spathaspora* sp. JA1, and *Wickerhamomyces anomalus* 740 for xylitol production from sugarcane bagasse hydrolysate under oxygen-limited conditions. The highest xylitol yield (0.83 g xylitol/g of xylose) was recorded from *W. anomalus* 740, but *M. guilliermondii* strain was able to tolerate the acidic environment and grows well even in the presence of 6 g/l acetic acid [42].

Xylose is taken up by cells with the help of specific or non-specific sugar transporters depending on the microbe, e.g., *Candida* species uses two types of transporter system acting separately depending on the physiological environment: under favorable conditions, cells uptake xylose via carrier-mediated facilitated diffusion transporter, while proton symporter operates under nutrition depleted conditions. Contrary to *Candida* spp., no such separate xylose-specific transporters have been reported from *Saccharomyces cerevisiae*, which imports xylose by both high- as well as low-affinity glucose transporters [43]. The final yield of xylitol was lowered due to the simultaneous utilization of xylose in central cellular metabolism for microbial growth and energy generation along with xylitol production. However, xylitol yield can be improved by supplementation with other carbon sources such as glucose or glycerol [14].

Enzymatic conversion is another way of biotransformation to avoid substrate loss in microbial counterparts. The use of xylose reductase for biotransformation of xylose allows to increase product yield and avoid substrate loss. Jang et al. designed an enzymatic transformation system by coupling NADH-dependent xylose reductase (XR) catalyzed reaction with formate dehydrogenase (FDH), which regenerates NADH/NADPH. The coupled reaction offers almost complete conversion (>99%) of xylose [44]. In another work, Walsh et al. reported 86.64% of substrate conversion with 7.92 U of xylose reductase, extracted from *Candida guillermondii* after 8 h [45]. It is evident that among the three approaches mentioned above, the enzymatic conversion is the most efficient and target-specific, with product yield up to 90–99% and can be performed under mild operating conditions in contrast to chemical catalysis. However, investment of an equivalent amount of NADPH/NADH for the conversion is a critical factor for process development.

### 2.4. Fourth Generation (Photo-Autotrophic Microbes)

Photoautotrophs are organisms that produce complex organic compounds (such as carbohydrates) using carbon di-oxide and acquire energy using photons. The ability to perform photosynthesis and reducing the powers enables them an attractive host for biochemical reduction compared to cell-free and heterotrophic systems. Cyanobacteria, which are the oldest group of photosynthetic organisms on the Earth with the ability to sustain harsh habitat conditions, attracted the researcher’s attention to use them as a suitable host. However, cyanobacteria are either devoid of natural xylose transport and reduction system or it exist in a suppressed form. To facilitate the xylose transport, xylose/proton symporter Ec-XylE and ATP-dependent transporter Ec-XylFGH were overexpressed in *Synechococcus elongatus* PCC 7942 [46] and *Synechocystis* PCC 6803 [47], respectively. These transporters enabled the uptake and subsequent utilization of xylose in cyanobacteria. Most recently, xylose transporter from *Escherichia coli* (Ec-XylE) and the NADPH-dependent xylose reductase from *Candida boidinii* (Cb-XR) was expressed in *Synechococcus elongatus* PCC 7942. Imported xylose was reduced by reducing power generated during photosynthesis yielding up to 0.9 g xylitol/g of xylose. It has been observed that xylitol productivity increased linearly with the cyanobacterial biocatalyst concentration and that the photosynthetic NADPH supply is sufficient for the reduction of xylose even under limited light penetration [40].

XR from a fungus *Neurospora crassa* was expressed in chloroplast genome of *Chlamydomonas reinhardtii* for the development of cost-effective microbial cell factory to produce biofuels and xylitol. Gene expression under 16S/atpA promoter/5 -UTR fusion exhibited the accumulation of heterologous protein and xylitol production with quite low yield (0.05 g/g xylose (0.38 g/L), suggesting possible improvement in productivity after further investigation [41]. Later, Zheng et al. found an inducible xylose transport system in *Chlorella sorokiniana* for the first time whose activity depends upon the other sugar molecules. Xylose uptake kinetics in *C. sorokiniana* under induced and non-induced state revealed that xylose uptake by cells is suppressed in the presence of D-glucose, D-galactose, and D-fructose, but not L-arabinose and D-ribose. The algal cells were grown in glucose, harvested and further grown in xylose. Remarkable increase in xylose uptake was recorded with D-glucose-induced algal cells at maximum transport rate of 3.8 nmol/min mg dry cell weight (DCW). In addition, xylose consumption was two times higher when grown in light in comparison with growth in the dark. Xylose uptake also activates associated metabolic enzymes, i.e., xylose reductase and xylitol dehydrogenase [48].

## 3. Enhancement in Production

### 3.1. Strain Improvement

Xylitol productivity can be increased by genetic modification of microbes to potentially replace the chemical process, as it can save energy and substrate purification costs. There are several parameters to be considered before designing the gene manipulation on microbes: genetic stability, regulatory and safety considerations, growth kinetics, expression levels of recombinant protein, good productivity, high yield and finally ease of product recovery. These parameters can be manipulated by (a) transport optimization, (b) xylose reductase over expression, (c) cofactor supply and engineering, (d) gene deletion and (e) several bioprocess parameters. Herein, we only mention a few key outcomes of these approaches as detailed revision of each one of these processes is beyond the scope of this review [49].

The recombinant strain of *Debaryomyces hansenii* was constructed by disrupting xylitol dehydrogenase gene, *xdh* improving the xylitol accumulation by almost 2.5-fold [50]. *Escherichia coli* strains were prepared by expressing a novel D-arabitol dehydrogenase-encoding gene (*ardh*) and xylitol dehydrogenase encoding gene (*xdh*), respectively, from *Gluconobacter* sp. JX-05. Co-biotransformation with both recombinant strains improved the xylitol yield by two times in comparison with wild *Gluconobacter* sp., possibly due to the utilization of other carbon sources along with xylose [51,52,53]. An endogenous aldose reductase gene *GRE3* and a xylose transporter gene *SUT1* gene were overexpressed in the industrial yeast strain of *S. cerevisiae* for xylitol production from agricultural biomass. Another gene, “G418 resistance marker cassette KanMX,” was also integrated into the yeast genome. The developed strain was evaluated for xylitol production from corn cob hemicellulosic hydrolysate containing glucose as the cosubstrate. *S. cerevisiae* (XP-RTK) exhibited higher xylitol productivity (212 mg/L h) than the control strain XP (81 mg/L h). Furthermore, the replacement of glucose by glycerol as a cosubstrate increased xylitol productivity to 318.6 mg/L h (47 g/L); however, the conversion factor for glycerol (0.47 mol/mol) was also lower than for glucose (23.7 mol/mol) [54]. Later, an engineered strain of *Saccharomyces cerevisiae* (YPH499-XR-BGL-XYL-XYN) was developed by expressing cytosolic xylose reductase (XR) along with β-D-glucosidase (BGL), xylosidase (XYL) and xylanase (XYN) enzymes co-displayed on the cell surface from various fungal and yeast resources. Cumulative expression of all these enzymes contributed to bioconversion of kraft paper pulp to xylitol with a conversion rate of 28% after 96 h. Moreover, the use of multiple enzyme systems significantly reduced the need for enzymatic pretreatment (commercial hemicellulase cocktail). A further expression of XYL and XYN on the cell surface using a SED1 “SSS” cassette improved the xylitol yield by 44% [55].

### 3.2. Alternate Substrates

Agriculture is an integral part and major source of raw material for most of the industries. These industries and agriculture itself generate huge amounts of residual waste, which is dominated by organic bio-molecules such as cellulose, hemicellulose and lignin (Table 4) [56].

**Figure 3:**
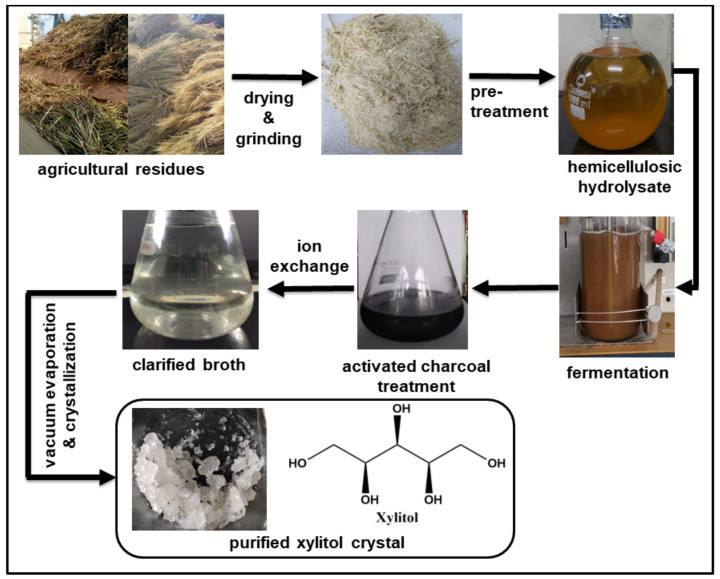
Processing of lignocellulosic biomass for xylitol production. Agricultural residues are dried and grounded to obtain coarse powder, which is treated with chemical/biological reagents to obtain hemicellulosic hydrolysate. Microorganisms ferment the hydrolysate to various byproducts such as xylitol. After fermentation, broth is treated with activated charcoal and ion exchange resins to remove the contaminants, and xylitol crystals are obtained after lyophilization of clarified broth.

Among developing countries, the majority of the waste is left unaddressed or burned. This unmanaged natural organic waste is rich in carbohydrate fraction, and thus represents enormous opportunities for industries if handled with scientific intervention [64,65]. Sugar fraction present in lignocellulosic residual biomass (LCB) from agriculture (e.g., sugarcane bagasse, corn stover, wheat straw, rice straw and rice hulls) and allied industries (e.g., pentose sugar/C5 rich waste from pulp and paper, food waste and pomace from juice and spent grain from beverages industries) can be utilized for the production of various value-added products such as xylitol (Figure 3) [10,64,66].

However, microbes and enzymes cannot directly access the sugar fraction due to the complex structure of lignocellulosic biomass and the presence of lignin and polyphenols. Various physical, chemical and biological treatments (Table 5) have been implemented for the removal of lignin, depolymerization of biopolymers and release of sugars for fermentation [67].

Pretreatment of lignocellulosic biomass produces pigments, acid-soluble lignin, phenols and other compounds, which hinder the microbial as well as enzymatic activity. Therefore, pretreatment may or may not be accompanied by detoxification by activated charcoal, ion-exchange chromatography or sodium boro-hydride to remove these inhibitors in post-hydrolysis processing [36,84].

## 4. Downstream Processing

Purity is an essential criterion for drug/nutraceuticals compounds. Fermentation broth contains numerous impurities like colorants, phenolic compounds, acetic acid, aromatic compounds and cell debris, which must be removed to obtain high-quality end-product. The selection of a cost-effective purification strategy for xylitol purification is equally vital as fermentation. Some of the standard methods used for xylitol recovery are discussed below.

### 4.1. Using Activated Charcoal and Ion Exchange Resins

Hemicellulosic hydrolysate has various undesired products such as colorants, inorganic salt, acetic acid, furfural, and hydroxymethylfurfural. These compounds may inhibit fermentation and affect product yield; therefore, it must be removed before or after fermentation. For this purpose, the fermentation broth is mixed with activated charcoal, followed by filtration. The process is quite effective for broth clarification and removal of colorants. Besides activated charcoal, ion-exchange resins (both cation-exchange resins and anion exchange resins in a sequential manner) are also used to remove ionic/charged impurities. Anion exchange resins are used for anionic colored compounds while the cation-exchange resins for desalination and removal of positively charged organic compounds. Activated charcoal treatment together with ion exchange resins is an efficient and economical way to eliminate the majority of contaminants [36,85].

### 4.2. Biphasic Extraction

Liquid-liquid extraction is a fast and straightforward process to eliminate unwanted impurities based on their solubility. However, effective separation needs the selection of an appropriate solvent. Earlier, ethyl acetate was found useful for both the clarification of fermented broth and purification of sugars and xylitol [6]. Mun et al. optimized the xylitol extraction from fermentation broth by response surface methodology and reported up to 79% recovery with ethyl acetate after 60 min with 1:4.5 solid liquid ratio [86]. In another work, supercritical fluid was used for xylitol recovery from the filtered broth. The broth was concentrated in a rotary evaporator at 75 °C, 280 mbar and 50 rpm followed by freezing at −20 °C. The concentrated sample was treated with supercritical CO_2_ in the presence of ethanol as a co-solvent. The process exhibited a maximum xylitol extraction efficiency of 40.51% when treated at the highest sample/solvent (11/9) ratio for a minimum extraction time of 20 min. The yield was quite low, but the purity of the obtained xylitol was greater than 89% [87].

### 4.3. Using Membrane Technology

Nanofiltration (NF) membranes separate molecules based on the difference in the particle size. Faneer et al. fabricated a Polyethersulfone (PES) NF membrane and evaluated the operating parameters for membrane performance in separating xylitol from other impurities and sugars. Nano-filtration with PES membrane retained more than 90% of xylitol from fermentation broth along with minor fraction of sugars as well. Repeated application of the process improves the purity of the end product [88].

Desiriani et al. also evaluated a membrane system for xylitol recovery from fermentation broth and removal of unwanted by-products (e.g., metabolic products, residual substances, biomass cells and mineral salts). A dual membrane separation system was developed with polysulfone ultrafiltration and polyamide nanofiltration (NF). Fermentation broth of *Debaryomyces hansenii* was filtered through the filtration system. Ultrafiltration rejected cell biomass almost completely while nanofiltration eliminates low molecular weight products such as acetic acid with very high retention potential for xylitol and negligible loss of sugar. Moreover, the two-stage separation process concentrated the xylitol by three times [89].

These results suggested the excellent performance of the membrane separation system for xylitol recovery. Nevertheless, its application at a large scale is questionable due to selectivity, handling of a large volume of fermentation broth and design, operating and membrane replacement cost. It was also found that membranes tend to retain sugars along with xylitol; therefore, additional processing is needed to remove the sugar fraction.

## 5. Application of Xylitol

### 5.1. Food Industry

**Figure 4:**
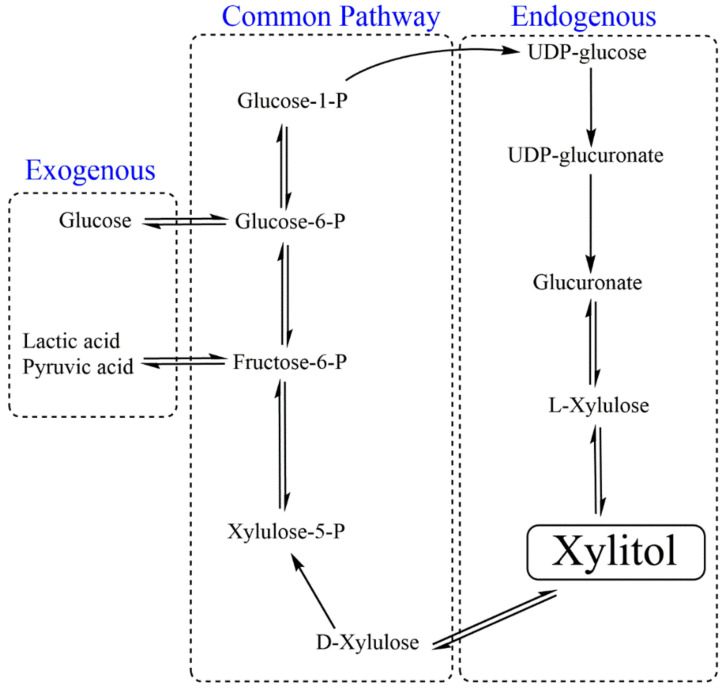
Metabolism of xylitol. Food and intracellular metabolism are two sources for xylitol. Xylitol consumed through food is referred to as exogenous xylitol and intracellularly-produced xylitol is endogenous xylitol. As shown in the figure, xylitol from both sources shares some common steps in metabolism.

Xylitol has an equivalent sweetness to sucrose; hence, its prime use in food products is as a sugar substitute. The nutritional value of food relies on its biological and chemical stability. Browning/Maillard reaction between reducing sugars and amino acids/proteins plays a crucial role in the chemical stability of food and imparts unique fragrance and taste to bakery products (Figure 4).

However, in some cases such as infant food, browning reaction is not recommended, as it reduces the food quality and nutrition value. Xylitol does not undergo browning reaction due to an absence of free aldehyde/ketonic groups. In addition, xylitol avoids microbial contamination as it acts as a sweetener-cum-preservative for food products. Thus, xylitol is preferred over other sweeteners for infant food formulation.

Another advantage of using xylitol as a sweetener in chewing gums, soft drinks, frozen desserts, confectionery, chocolates, jams, puddings and ice creams is the generation of a cool aftertaste (Table 6). This is due to high endothermic heat of solution (34.8 cal/g), which captures heat from surroundings and imparts a cooling effect [90,91,92].

Diabetes: Energy balance and controlled energy intake are matter of concern for obese individuals, diabetics and people with other metabolic syndromes. Hence, low-calorie substitutes such as saccharin, aspartame, neotame, sucralose or acesulfame potassium are preferred to sugar in order to avoid hyperglycemia. Prolonged consumption of synthetic sweeteners also exhibited detrimental or life-threatening side effects such as gastric, pancreatic, endometrial cancers, lymphomas, migraines, fibromyalgia, reduced anti-oxidation potential of the liver and genetic diseases [93]. Metabolically, xylitol plays a dual role: as a sweetening agent and as an energy provider with better anti-catabolic action (insulin resistance, e.g., postoperative and posttraumatic states); thus, it is also referred to as a nutritive sweetener [94]. Early metabolic fate of exogenous (consumed from external sources) and endogenous (synthesized within cells during uronic cycle) xylitol is different (Figure 4) [95]. In contrast to other cells, hepatic cells are highly permeable and metabolically active for xylitol. Exogenous xylitol reaches to intestine and uptake by intestinal mucosa by passive or facilitated diffusion. The absorption of xylitol is still much slower than glucose [96,97].

Theoretically, hepatic cells metabolize xylitol via two possible routes: first, it is oxidized to D-xylulose by a nonspecific NAD-linked polyol dehydrogenase, and to L-xylulose in the presence of NADP-linked polyol dehydrogenase. Kinetic study of both the enzymes revealed that the production of D-xylulose is preferred over L-xylulose. From here, both endogenous and exogenous xylitol follows the same metabolic pathway. D-xylulose kinase phosphorylates D-xylulose to D-xylulose 5-phosphate, which is converted to fructose-6-phosphate and glyceraldehyde-3-phosphate. These molecules are intermediates as well as connecting links to glycolysis and gluconeogenesis, which lead to pyruvate and lactate production from glucose and glycogen, respectively. After xylitol consumption, blood glucose level does not respond much, as gluconeogenesis predominates glycolysis. Its concentration in the blood remains low even after oral administration due to its slow absorption and rapid metabolism in liver cells [98].

Islam and Indrajit analyzed the antidiabetic effects of xylitol in the type 2 diabetes rat model in three groups: normal control, diabetic control and xylitol. Xylitol has contributed to diabetes management by inhibiting the carbohydrate metabolizing enzyme, slowing down the absorption of glucose in the intestine and promoting the same in skeletal muscles. Xylitol consumption results in lower body weight, blood glucose and serum fructosamine after five weeks of intervention in the xylitol group [99]. Besides the inhibition of carbohydrate hydrolyzing enzymes such as α-amylase and α-glucosidase, xylitol also delays the absorption of glucose in duodenal and jejunal segments of intestine and improves the uptake of circulating glucose by muscle tissue [100,101].

### 5.2. Pharmaceutical Industry

The biological potential of xylitol has been a topic of research interest recently. Xylitol consumption reduces plaque levels, xerostomia, gingival inflammation and nasopharyngeal pneumonia, and hence, is a valuable product for pharmaceutical industry. It reduces the microbial load by multiple mechanisms including anti-adhesive, oxidative stress, low permeability and futile metabolism [102,103,104,105].

#### 5.2.1. Oral Hygiene and Dental Caries

Otopathogens produce acid from the sugar fermentation, which demineralizes teeth. Xylitol improves salivary flow, raises saliva pH and suppress the growth of otopathogens such as *Streptococcus* mutants and *Helicobacter pylori* [102,104]. The effect of xylitol concentration on subgingival plaque and cariogenic and periodontal bacteria was evaluated. Two chewing gums were selected with 100% and 22% xylitol content, respectively, and saliva concentration of xylitol and corresponding microbial growth was recorded in 32 subjects before, while chewing, and after discarding the gums. Reduction in bacterial load followed the same pattern, which increases with the concentration of xylitol. The experiment revealed that xylitol could reduce the cariogenic and periodontal bacterial load [106].

Presence of bioactive molecules further improve the antimicrobial potential of xylitol due to a synergistic effect. For this purpose, three types of chewing gums were prepared with Xylitol, Polyols (Pols) and Magnolia (Xylitol + Magnolia) and evaluated against 271 high-risk subjects for caries lesions, gingival bleeding, *Streptococci mutant* and plaque pH after two years. Caries lesions, gingival bleeding, plaque and *Streptococci mutants* load were significantly lower in the subject group kept on chewing gum containing xylitol and Magnolia in comparison to xylitol alone [107]. Rebaudioside-A is a natural steviol glycoside which is sweeter than sucrose. The effect of Rebaudioside-A on otopathogens was evaluated in vitro and compared with sucrose and xylitol. All three sweeteners were added to saliva separately, and microbial load of *Streptococcus mutans*, *Streptococcus sobrinus*, *Streptococcus oralis*, *Lactobacillus rhamnosus*, *Lactobacillus paracasei* and *Candida albicans* was recorded after 10 h in each case. Additionally, a change in pH was also determined. In comparison to sugar substitutes (xylitol and rebaudioside-A), the maximum pH drop was recorded in the sucrose group. However, xylitol was found superior to rebaudioside-A in reducing the microbial load of *Streptococci mutants* and other otopathogens. At the same time, rebaudioside-A is as efficient as xylitol in lowering acid synthesis, preventing a drop in pH and tooth demineralization [108]. Real-Time Cell Analyzer also revealed a reduction in viable cells of otopathogens such as *Streptococcus mutants* after regular consumption of xylitol. Lower levels of polysaccharide content and expression of genes involved in glucan-mediated biofilm formation, including *gbpB*, *gtfB*, *gtfC* and *gtfD*, were also detected. Xylitol was also found to prevent surface attachment of otopathogens by reducing biofilm formation [109].

#### 5.2.2. Respiratory Tract Infection

Xylitol’s antimicrobial potential has also been exploited for respiratory tract hygiene. It has been used in the formulation of airway surface liquids to lower the number of nasal coagulase-negative *Staphylococcus* by reducing the airway surface liquid (ASL) salt concentration and enhancing the innate antimicrobial defense in the respiratory pathway [110]. *Burkholderia cepacia* is a common pulmonary pathogen which prevails due to poor clinical practices during lung transplantation. *B. cepacia* complex infection is common in patients with cystic fibrosis. Treatment of human airway explants with xylitol (60–80 mg/mL) inhibited the growth of the *Burkholderia cepacia* complex (BCC) up to 65%, which is quite crucial for a successful lung transplant [111]. According to the World Health Organization (WHO), 650.000 casualties are caused by respiratory diseases annually, with the Influenza A virus being one of the leading causes among them. Antiviral activity of xylitol was examined by Yin et al., who studied the effect of xylitol and red ginseng extract on mice infected with H1N1 [112]. They have found that the administration of ginseng together with xylitol has a synergistic effect, increasing the survival rate of mice by ameliorating the influenza virus infection. In chronic rhinosinusitis (CRS), the effect of antibiotics is reduced/restricted due to biofilms formed by pathogens. Four subject groups were created, which were administered with 5% xylitol, 10% xylitol, saline solution and one without any treatment. The effect of the treatments was evaluated for CRS biofilm formation and growth of *Staphylococcus epidermidis*, *Pseudomonas aeruginosa* and *Staphylococcus aureus* by crystal violet assay. The microbial biofilm by *S. epidermidis*, *S. aureus* and *P. aeruginosa* was reduced significantly in groups administered with 5 and 10% xylitol in a concentration-dependent manner. However, 5% of xylitol was more effective in inhibiting biofilm formation by *S. epidermidis. S. aureus* biofilm was disrupted by saline, 5% xylitol and 10% xylitol. In contrast, *P. aeruginosa* biofilm was unaffected by any of the treatments [113].

Xylitol administration effectively reduced lung virus titer of human respiratory syncytial virus (hRSV), which causes bronchiolitis and pneumonia in infants. On the molecular level, CD3^+^ and CD3^+^CD8^+^ lymphocytes, responsible for inflammation, were quite low in the case of xylitol-administered group [114].

#### 5.2.3. Acute Otitis Media

Acute otitis media (AOM) is one of the most frequent recurrent childhood infections and can lead to additional complications such as mastoiditis, meningitis and impaired hearing due to an effusion-flooded middle ear. Xylitol reduces the adherence of AOM causing pathogens, e.g., *Streptococcus pneumoniae* and *Haemophilus influenzae* to nasopharyngeal cells by 40%. Xylitol was effective against both antibiotic-sensitive and -resistant diplococci *Streptococci*, independent of the patient’s immunity. It was quite effective in curing pneumonia and reducing the mortality rate in neonates [115]. The possible mechanism behind its antiadhesive effect against the otopathogens is its ability to manipulate the structure of bacteria [87] and blocking of bacterial lectins [116].

#### 5.2.4. Hemolytic Anemia

**Figure 5:**
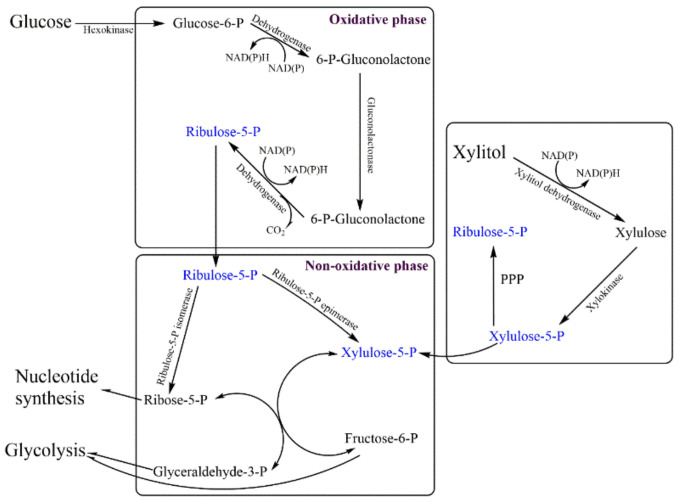
Compensation of NADPH deficiency from xylitol metabolism by Hexose monophosphate to counter oxidative stress in glucose-6-phosphate deficiency induced hemolytic anemia. Due to glucose 6 phosphate deficiency, cells are unable to recover from oxidative stress due to a shortage of NADPH_2_. Xylitol metabolism aid in generation of cofactor.

The hexose monophosphate (HMP) shunt is a crucial metabolic pathway for cell functioning and survival. It not only generates various intermediates/precursors for different biomolecules, including nucleic acid and proteins, but also recovers cells from oxidative stress and reactive oxygen species. An HMP shunt generates NADPH_2_ to recover cells from oxidative stress and facilitates the reduction of glutathione to stabilize the cell membrane. HMP shunt operates in a two-phase process: the oxidative phase, in which NADPH_2_ is generated, and the non-oxidative phase/Pentose Phosphate Pathway (PPP), generating precursor biomolecules. Glucose-6-phosphate dehydrogenase (G6PD) catalyzes the NADP reduction and counterbalances the oxidative stress. In malaria-endemic areas, deficiency of G6PDH is common due to the consumption of oxidizing chemicals/antimalarial drugs, and the condition eventually results in hemolytic anemia. Since mitochondria are absent in red blood corpuscles (RBCs), the pentose phosphate pathway (PPP) is the only defense against oxidative damage by generating NADPH (Figure 5).

G6PD deficiency is an X-chromosome linked genetic defect, which leads to neonatal jaundice and acute hemolytic anemia. The presence of NADP-linked xylitol dehydrogenase (XRD) has been reported in normal as well as G6PD-deficient RBCs of humans. Xylitol dehydrogenase generates NADH to counter oxidative stress and could possibly be used for the treatment of G6PD-deficient hemolytic anemia as well as in prevention of acetyl-phenylhydrazine-induced acute hemolysis of rabbit RBCs [117,118,119].

#### 5.2.5. Anti-Cancerous and Anti-Inflammatory Activity

Cancer is the uncontrolled growth of cells, which may be in many cases caused by microbial infection triggered chronic inflammation. Treatment with anti-inflammatory agents reduces the severity of inflammation and the risk of cancer [120]. *Porphyromonas gingivalis* induces inflammation due to the production of proinflammatory cytokines, including tumor necrosis factor-α and interleukin (IL)-1β, release of cytokines and chemokines, e.g., IL-12, p40, eotaxin, interferon γ–induced protein 10, and monocyte chemotactic protein-1 and macrophage inflammatory protein-1 expression. Xylitol inhibits the adhesion of *P. gingivalis* on THP-1-derived macrophages and suppresses the production of cytokines, nitric oxide and exerted antiphagocytic activity against both *P. gingivalis* and *Escherichia coli* [14]. Usually, chemotherapeutic agents are used for primary cancer treatment. Unfortunately, these chemicals affect the normal cells as well, resulting in various side effects, including hair loss, nausea and neurotoxic side effects, even on healthy cells. On the contrary, xylitol can inhibit the proliferation of different cancer cells, e.g., A549, Caki, NCI-H23, HCT-15, HL-60, K562 and SK MEL-2. The half maximal inhibitory concentration (IC50) of xylitol and its specificity was found to be higher in human gingival fibroblast cells. It also induces autophagy in A549 cells and inhibits the proliferation of A549 cells [121,122]. The effect of xylitol and glycerol was also evaluated against osmotic stress in keratinocytes “HaCaT keratinocytes.” Different concentrations of both polyols were used: 0.027%, 0.27% and 0.045%, 0.45% for glycerol and xylitol respectively, and resultant cellular viability, cytotoxicity, intracellular Ca^2+^ concentration and expression of RNA related to inflammatory cytokines were observed. Both polyols are effective in countering the hyperosmotic stress, however, their mechanism and efficacy varies as glycerol improves the cellular viability and suppresses the expression of IL-1α, IL-1β and NFAT5, while xylitol acts by suppressing the expression of IL-1α and preventing the rapid Ca^2+^ signal [123].

#### 5.2.6. Cardiovascular Diseases and Lipid Metabolism

Visceral obesity is the origin of many acute and deadly health ailments such as metabolic syndrome, atherosclerosis, cardiovascular disease, stroke and even cancer. Xylulose-5-phosphate activates carbohydrate response element-binding proteins and transcription of associated lipogenic enzymes. In rat model with visceral fat mass, plasma insulin and lipid concentrations were significantly lower than in control subjects. Xylitol increased the gene expression of lipogenic enzymes, fatty acid oxidation-related genes and carbohydrate-response element-binding protein (ChREBP), while the expression of sterol regulatory element-binding protein 1c was suppressed [121,124]. It was also reported that xylitol consumption improves the tolerance of various organs, including the heart, liver, kidney and pancreas, against diabetes-associated oxidative stress. Xylitol proves to be useful as more than a sweetener for diabetics and cardiac patients [101].

#### 5.2.7. Osteoporosis

Osteoporosis, a type of systemic skeletal diseases, occurs due to an increase in bone resorption, which is manifested by low bone mass, microarchitectural deterioration of bone tissue and fragile bone. Similarly, the imbalance between bone resorption and bone formation leads to osteopenia. As of now, no efficient and safe method is available to prevent either disease. Bone mass loss due to menopause, aging, genetic makeup, nutritional habits and lifestyle are some of the factors affecting peak bone-mass directly. Treatment with estrogens, bisphosphonates and calcitonin may be used to reduce/prevent osteoporotic bone loss. Dietary supplementation of xylitol increased calcium and phosphorus levels in bones, promoted the restoration of bone calcium, and protected against the ovariectomy-induced loss of bone minerals during experiments featuring rats with artificially induced osteoporosis [125,126].

### 5.3. Application in Other Industries

#### 5.3.1. Personal Care

Besides other mechanisms, low transepithelial permeability is also responsible for antimicrobial potential of xylitol against skin pathogens. Its efficiency was further increased in combination with chlorhexidine [127]. Xylitol was also evaluated for its hydration and antimicrobial potential against skin-associated bacteria, including *Staphylococcus aureus*, *Staphylococcus epidermidis* and *Cutibacterium acnes*. It was reported that xylitol had both growth-inhibiting and growth-promoting effects on pathogenic microbes in a concentration-dependent manner. *S. epidermidis* growth was promoted at 1% xylitol, but 5% xylitol inhibited the growth of *S. aureus*, *C. acnes* and *S. epidermidis* as well. The results suggested the possible use of xylitol in the formulation of personal care products [128].

#### 5.3.2. Biopolymer Synthesis and Tissue Regeneration

In recent years, xylitol has also been used for the synthesis of biodegradable and biocompatible polymer matrices and fibers. Certain types of cross-linking processes, parameters and components involved are responsible for different physical and chemical properties of polymers. Two xylitol-containing polymers, i.e., “poly (xylitol succinate-co-butylene succinate) PXBSu” and “poly (xylitol sebacate-co-butylene sebacate),” were developed by copolymerization of sebacic acid: butylene glycol: xylitol and succinic acid: butylene glycol: xylitol, respectively. The developed polymers exhibited improved mechanical strength and other physical properties [129]. Similar kinds of efforts were made to control the physical and chemical properties of xylitol-based polymer poly (xylitol-dicarboxylate-co-butylene dicarboxylate) using five different dicarboxylic acids: adipic acid, dodecanedioic acid, sebacic acid, succinic acid and suberic acid. The samples were withdrawn at definite intervals during the polycondensation process. After screening different pre-polymers, the optimum results were obtained after 288 h of the polymerization reaction. It was also found that dicarboxylic acid chain length has a direct influence on the physical and chemical properties of the polymer [129]. Diverse characteristics of biopolymers also direct their applications in different sectors, including healthcare. For repair and regeneration of tissues, the activity of fibroblast growth factor (FGF) is critical. Its clinical applications are under threat due to its low stability, short half-life and rapid inactivation by enzymes. FGF was loaded on a xylitol-based polymer poly (xylitol dodecanedioic acid) (PXDDA) by a simple dopamine coating method. PXDDA-FGF improved and regulated the release of FGF and enhanced the human fibroblast cells attachment and proliferation. The results indicated the bright future of tissue regeneration with biocompatible polymers [130].

## 6. Side Effects

Unlike glucose and galactose, xylitol follows a differential pattern for absorption and metabolism. A significant portion of xylitol is absorbed in the small intestine and a small fraction reaches the colon. Here, colonic microbiota ferment it to produce short-chain fatty acids, which ultimately serve as energy and strengthen the host immune system. The presence of excess amounts of xylitol increases the osmotic pressure of digesta reaching colon. As a result, a large amount of water is retained in digesta, exhibiting laxative effects. Xylitol is metabolized or absorbed completely and does not reach colon. Initially, cells are not adapted to xylitol metabolism, and sudden consumption of large amounts of xylitol and its movement towards the colon can result in osmotic diarrhea. A similar mechanism operates in rats as well, but in dogs, the absorption is much faster and complete, which reflects in blood. At the preliminary stage, the low diet of up to 50 g/day is suggested; however, regular consumption helps in uplifting the tolerance level without any adverse effects. On the contrary, it is a life-threatening toxin for dogs, causing hypoglycemia and osmotic diarrhea because of different metabolism patterns [5,101,131,132,133].

## 7. Conclusions

Xylitol holds a significant market share in sweeteners, but the conventional chemical catalysis and enzymatic conversion face the bottleneck of substrate limitation and high cost of production. Bio-production using microbes might be cost-effective for xylitol production, but its application at a large scale is questionable due to the unstable expression and variable yield. Challenges can be tackled with the implication of more sophisticated tools, such as genome editing and mutagenesis, which help in the development of strains with higher conversion rates and tolerance for inhibitors. Moreover, designing an integrated process with multiple products and cost-effective recovery processes further facilitates to reduce product cost. Support for nanotechnology and material sciences improves the efficiency of traditional applications such as sweetener alternatives, anticarcinogenic and antimicrobial drugs.

Xylitol holds a significant market share in the food and pharmaceutical sector, especially in sweeteners and other pharmaceutically important products. Although chemical catalysis may be sufficient to fulfill the market requirement, high cost of production and polluting chemical residues further deteriorates the environment. Process modification is the ultimate way out for lowering the overall cost, besides addressing the environmental concerns. Therefore, the use of hyperproducer microbes, alternate low-cost substrates and efficient recovery processes are of the utmost significance. The microbial and enzymatic processes have emerged as an efficient and cost-effective approaches for xylitol production. Still, their commercial utilization is hindered due to the simultaneous degradation by microbes and substrate specificity for enzymes. Therefore, the biological approach is promising for the large-scale production of useful products such as xylitol. Screening of microbial community, strain improvement and process development involving alternate low-cost substrates such as lignocellulose are essential areas with immense future possibilities. Genetic modifications and genome editing may further help in enhancing the xylitol yield by increasing the expression of xylose reductase and other enzymes for xylitol production from sugar moieties, other than expensive substrates such as xylose, and blocking the xylitol metabolism. On the one hand, these efforts will help to explore microbial diversity; on the other, they will provide inexpensive and safer food and pharmaceutical products while simultaneously addressing the environmental concerns.

## Figures and Tables

**Table 1:** Leading manufacturer of xylitol and its production overview in metric tons, as updated on the respective company websites.

Manufacturer	Country	Substrate	Metric Tons/Year
Futaste pharmaceutical Co., Ltd.	China	Corn cobs	31,000
Shandong Lujian Biological Technology Co., Ltd.			16,000
Anhui elite industrial Co., Ltd.			5400
Hefei reachever import and export limited company			5400
Hunan JK international trade corporation			12,000
Shanghai just import and export Co., Ltd.			55,000
			5000
Hangzhou Shouxing Biotechnology Co., Ltd.			4000
Shandong Biobridge Technology Co., Ltd.			6000
Tangyin Hung Industrial Co., Ltd.			2500
Thomson Biotech (Xiamen) Pte., Ltd.			10,000
Yucheng Lujian Biological Technology Co., Ltd.			6000
Zhejiang Huakang Enterprise Co., Ltd.			20,000
Shijiazhuang Acid Chemical Co., Ltd			10,000
Shengquan Healtang Biotech Co., Ltd.			8000
Xylitol USA, Inc	USA	Birch trees	-
DuPont (Danisco) USA		Waste side stream of a pulp and paper plant to extract xylose	2000
Superior Supplement Manufacturing		-	-
Avansecure	India	Corn husks, sugar cane bagasse and birch	-
Salvavidas			-
Herboveda			-
Geno Chem, Ltd			-
Leisha Pharma Solutions Pvt., Ltd.			-

**Table 2:** Xylitol concentration in fruits and vegetables [6,8].

Product	Xylitol Content (mg/100 g Dry Weight)
Carrot juice	12
Chestnut	14
Banana	21
Carrot	86.5
Onion	89
Lettuce	96.5
Pumpkin	96.5
Spinach	107
White mushroom	128
Eggplant	180
Raspberry	268
Cauliflower	300
Strawberry	362
Yellow plum	935
Lingon berry	64
Cran berry	37
Bilberrya	38
Sea buckthorn	91
Rowan berry	160
Apple	128

**Table 3:** Xylitol production by different microorganisms, and their productivity using different xylose sources.

Organisms	Substrate	Yield (Y_P/S_) and Productivity	References
**Bacteria**			
*Bacillus subtilis*	Xylose	0.85 g/g xylose (213 g/L)	[24]
*Escherichia coli*	Xylose	0.612 g/g xylose (6.325 g/L)	[25]
*Cellulomonas cellulans* NRRL B-4567	Xylose	1.76 g/L xylitol, 1.67 g/L ethanol	[22]
*Mycobacterium smegmatis*	D-xylulose, D-mannitol	0.7 g/g xylulose	[26]
**Fungi**			
*Aspergillus niger*	D-glucose and D-xylose	0.211 g/g biomass (1.139 g/L)	[27]
*Trichoderma reesei*	Barely straw	0.122g/g biomass (6.1 g/L) and 26.44 g/g biomass (13.22 g/L)	[28]
*Thermomyces lanuginosus* SSBP	Xylose from sugarcane bagasse	0.22 g/g xylose (4.4 ± 0.13 g/L)	[29]
**Yeast**			
*Meyerozyma guilliermondii*	Xylose	0.27 g/g xylose (4.28 ± 1.30 g/L)	[30]
*Debaromyces hansenii* UFV-170	Xylose	0.73 g/g (76.6 g/L)	[31]
*Debaryomyces nepalensis* NCYC 3413	Xylose + Glucose	0.54 g/g (48.6 g/L)	[32]
*Hansunela anomala* NCAIM Y.01499	Xylose	0.174 g/g xylose (8.7 g/L)	[33]
*Saccharomyces cerevisiae*	Pretreated corn stover	0.99 g/g-consumed xylose (45.41g/L xylitol) and 50.19g/L ethanol	[34]
*Saccharomyces cerevisiae*	Wheat stalk	3.47 g/L	[35]
*Pachysolen tannophilus*	Brewer’s spent grain	0.47 ± 0.06 g xylitol/g xylose and 0.09 ± 0.002 g ethanol/g xylose	[36]
*Scheffersomyces amazonensis* UFMG-HMD-26.3	sugarcane bagasse and straw hemicellulose hydrolysate	0.5 g/g xylose (28.56 g/L)	[19]
*Kluyveromyces marxianus* CCA510	Cashew apple bagasse	0.50 g/g (6.01 g/L)	[20]
*Pachysolan tannephilus* ATTC 32691	Xylose	0.14 g xylitol/g and 0.39 g ethanol/g	[37]
*Saccharomyces cerevisiae*	Xylan	0.71 g/g xylan (1.94 g/L)	[38]
*Kluyveromyces marxianus* IIPE453t	Sugarcane bagasse	0.42 g/g biomass (25.6 g/L)	[39]
**Cyanobacteria and Algae (Photoautotrophs)**			
*Synechococcus elongatus* PCC794	Xylose	0.85 g/g (33 g/L)	[40]
*Chlamydomonas reinhardtii* (expressing XR from *Neurospora crassa*)	Xylose	0.05 g/g xylose (0.38 g/L)	[41]

**Table 4:** Major agricultural residues generated annually in different countries.

Country	Major Crops-Residues Fraction	Agricultural Waste Generated (≈Million Tons/Year)	References
India	Rice, wheat, sugarcane, maize	500	[57]
Bangladesh	Maize, rice	72	[58]
Indonesia	Rice, maize	55	[57]
Myanmar	Rice	19	[57]
China	Rice, sugarcane, maize, soybean	930.8	[59]
Pakistan	Wheat, sugarcane, rice	40	[60]
Brazil	Sugarcane, maize	597	[61]
Malaysia	Rice	1.2	[62]
Nigeria	Barley, maize	145.62	[63]

**Table 5:** Different pre-treatment strategies used for biomass processing and sugar recovery.

Pre-Treatment Strategies	Operating Conditions	Mechanism	References
**Physical**			
Milling and grinding	Drying, milling to fine or coarse powder	More surface area, improve flow properties, increase the bulk density and porosity	[68,69]
Irradiation	γ-radiation and electron beam	Scission of glycosidic bonds in polysaccharides and destruction of the cell wall	[70,71]
**Physico-chemical treatment**			
Autohydrolysis and steam explosion	160–260 °C and 5–50 atm pressure 1% acid may be added	The complex structure of LCB is disrupted due to the expansion of steam	[72,73]
Microwave radiation (MWR)	MWR/water, MWR/alkali, MWR/acid, MWR/ionic liquid, MWR/salt	Accelerates cellulose dissolution in ionic liquids, removes hemicellulose and lignin	[74]
**Chemical treatment**			
Acid	CH_3_COOH, HCl and H_2_SO_4_ (Dilute or concentrated acid)	Disruption of the hydrogen bonds and covalent bonds, solubilization of hemicellulose and reduction of cellulose complexity	[75]
Alkali	KOH, NaOH, Ca(OH)_2_, Ammonia (ammonia fiber expansion)	Destruction of lignin, reduction of the degree of polymerization of hemicellulose, lower crystallinity of cellulose	[76,77,78]
Ionic liquids	1-butyl-3-methyl-imidazolium acetate, cholinium ionic liquid, etc.	Attachment of hydrogen bonds to dissociate the lignocellulose complex	[79]
**Biological treatment**			
Microbiological treatment	Yeast, fungi, micro-algae, bacteria	Enzymes break respective bonds and depolymerize/solubilize polymers	[79,80,81]
Enzymatic hydrolysis	Xylanases and cellulases		
**Nanotechnology in biomass pretreatment**			
Nanoparticles of metal/biopolymers	Acid/base/enzymes/microbes	Nanoparticles improve the delivery of agents and enhance the activity	[79,82,83]

**Table 6:** Xylitol based commercial products in food industry. The concentration of xylitol (%) used in the products are also listed.

Genre	Brand Name	Trademark	Concentration (%)
Chewing gum	Trident	Trident, USA	1
	Epic Dental	Epic, USA	1
	Xylitol Sugar Free Chewing Gum	Lotte, Thailand	-
	Xylitol Chewing gum	Hager Werken, Germany	-
Candies and drops	Xylipop	Hager Werken, Germany	-
	Xylitol drops	Hager Werken, Germany	94
	Xylitol candy	Ice Chips candy, USA	
	Xylitol	Epic, USA	1
	Xyla	Xylitol, USA	0.4
	Snowflakes	Snowflakes, USA	2
Mouthwashes and toothpastes	Spry mouth wash	Xlear, USA	-
	Act Braces Care	Chattem, USA	-
	TheraMints	3M, USA	1
	Xyli White	New Food Solutions, USA	25
	Bioxtra	Hetero Healthcare Ltd., India	-
	Bioxtra-T	Hetero Healthcare Ltd., India	-
Beverages	Lime refresher	Naturally sweet, Australia	6.6
	Citron tea	Yesan-nongsan Co., Ltd., Korea	-
	Honey	Health Garden, USA	10
	Xylitol Real birds nest	Scotch Real, Thailand	10
Sweetener	Xylitol alternative	Suganon, South Africa	-
	Xylitol plus	Now, USA	1.7
	So Sweet xylitol	Ankur drugs and pharma Ltd., India	-
